# Chemotherapy Before Radiotherapy in Adjuvant Breast Cancer: The Origins of a Convention and the Case for Revisiting Sequence in the Era of Hypofractionation

**DOI:** 10.3390/medsci14040477

**Published:** 2026-08-13

**Authors:** Mihai-Teodor Georgescu

**Affiliations:** Oncology 2 Discipline, Department 8—Radiology, Oncology, Hematology, Faculty of Medicine, “Carol Davila” University of Medicine and Pharmacy, Bulevardul Eroii Sanitari 8, 050474 Bucharest, Romania; mihai.georgescu@umfcd.ro; Tel.: +40-212271195

**Keywords:** breast cancer, adjuvant radiotherapy, treatment sequencing, hypofractionation, concurrent chemoradiotherapy, sandwich radiotherapy, locoregional control, adjuvant chemotherapy

## Abstract

Guidelines in early breast cancer place adjuvant chemotherapy before radiotherapy, permit radiotherapy concurrently with endocrine and anti-HER2 agents, but discourage it before or during cytotoxic chemotherapy. This narrative review asks whether that convention remains defensible. Its historical basis, a single randomised trial whose distant-metastasis signal did not survive long-term follow-up, is weaker than is commonly assumed. Its contemporary basis is stronger and is stated here explicitly: an asymmetry of absolute benefit, in which a two-point gain in locoregional control yields roughly half a percentage point of mortality benefit once the EBCTCG four-to-one relationship is applied, whereas degradation of systemic therapy reaches mortality undiscounted. We specify three conditions under which a sequencing change could be justified and note that the absolute loss from a short chemotherapy delay cannot presently be quantified from randomised data. Meanwhile, chemotherapy has lengthened while radiotherapy has contracted to a one- to three-week whole-breast schedule, potentially extended when a sequential tumour-bed boost is required. The retrospective evidence is limited: one cohort offers a hypothesis-generating locoregional signal qualified by an unexpectedly high control-arm event rate and an unplanned subgroup analysis, while another supports only short-term feasibility and tolerability; neither demonstrates benefit in distant control or survival. We further argue that the population in which the question remains clinically live is narrow and probably contracting, since genomic de-escalation withdraws from chemotherapy the phenotypes with the most favourable arithmetic. Adjuvant sequencing is best regarded as an open, testable question within a defined population rather than a general case for change.

## 1. Introduction

Two local and one systemic problem confront the clinician treating early breast cancer: residual disease in the breast or chest wall and regional nodes, and micrometastatic disease seeded beyond them. Radiotherapy and chemotherapy address these problems in largely separate compartments, yet their benefits are complementary, and for a large proportion of patients, both are indicated. Meta-analyses of individual patient data have established that radiotherapy after breast-conserving surgery roughly halves the ten-year risk of any first recurrence and reduces the fifteen-year risk of death from breast cancer, with the survival benefit approximately proportional to the reduction in local recurrence [1]. Adjuvant systemic therapy independently lowers the risk of distant relapse and death. When both modalities are warranted, the question is not whether to give them but in what order.

For early breast cancer, the answer has, since the mid-1990s, been remarkably uniform. Chemotherapy is delivered first; radiotherapy follows on its completion. Radiotherapy may be given concurrently with endocrine therapy and with anti-HER2 agents, and its concurrent use with immunotherapy is an area of active study, but it is not, as a rule, delivered before conventional cytotoxic chemotherapy or interleaved between its cycles, and this ordering is embedded in international guidelines [2,3]. The convention is so entrenched that it is often presented to trainees and patients as self-evident. It is not. It was adopted on the strength of limited evidence, has survived the maturation of that same evidence into a null result, and was framed around chemotherapy regimens and radiotherapy schedules that bear little resemblance to those in use today [2].

This review has a deliberately narrow focus. We set aside the neoadjuvant setting, in which systemic therapy necessarily precedes locoregional treatment, and concentrate on the adjuvant sequencing of chemotherapy and radiotherapy after definitive surgery. We first reconstruct the origins of the chemotherapy-first convention and the biological and clinical arguments marshalled in its favour. We then explain why endocrine and targeted agents are treated as compatible with concurrent irradiation while cytotoxic drugs are not. Finally, we examine how third-generation chemotherapy and hypofractionated radiotherapy have jointly destabilised the assumptions on which the convention rests, and we appraise the emerging evidence for sandwich and upfront radiotherapy. We acknowledge at the outset that the exclusion of the neoadjuvant setting is consequential rather than merely tidy: as higher-risk patients increasingly receive systemic therapy before surgery, the adjuvant population to which this argument applies is correspondingly narrower, a point taken up in Section 8. Our aim is not to overturn current practice but to argue that its central premise has become a testable hypothesis rather than an established fact.

## 2. Anatomy of a Convention: The Upfront–Outback Trial

The modern convention can be traced to a single randomised trial conducted at the Joint Center for Radiation Therapy, later known colloquially as the “Upfront–Outback” study. Two hundred and forty-four patients with stage I or II breast cancer at substantial risk of distant metastasis, all treated with breast-conserving surgery, were randomly assigned to receive a twelve-week course of chemotherapy either before or after radiotherapy [4]. At a median follow-up of just under five years, the group irradiated first showed a higher rate of distant metastasis than the group treated with chemotherapy first, 36% versus 25%, a difference in borderline statistical significance [4]. Local recurrence, by contrast, tended to be more frequent when radiotherapy was deferred. The investigators interpreted the distant-metastasis signal as the more clinically consequential of the two and concluded that, for patients at meaningful risk of systemic relapse, it was preferable to deliver chemotherapy before irradiation.

Two features of this trial are routinely underappreciated. The first is that its own long-term analysis did not confirm the early signal. When the cohort was re-examined with mature follow-up, no statistically significant difference remained between the two sequences in freedom from distant metastasis, freedom from any event, or overall survival, and toxicity was comparable [5]. The chemotherapy-first arm was nonetheless retained as the standard, a decision driven as much by mechanistic reasoning as by the data. The second is that the chemotherapy tested was a twelve-week regimen. The authors were explicit that their findings could not be extrapolated to the longer, multi-agent regimens then entering practice [4,5]. A convention that governs the sequencing of six months of modern chemotherapy therefore rests on a trial of twelve weeks of an older one, whose distant-metastasis signal did not survive the passage of time.

## 3. The Rationale for Chemotherapy-First

Beyond the historical trial, three interlocking arguments sustain the convention. They concern where mortality comes from, whether radiotherapy can safely wait, and what happens when the two modalities overlap.

### 3.1. Mortality Is a Systemic Problem

The dominant argument is one of competing hazards. Death from breast cancer is, in the great majority of cases, a consequence of distant metastatic disease rather than of uncontrolled locoregional recurrence. Systemic chemotherapy is the instrument that addresses occult micrometastases, and any delay in its delivery is therefore assumed to carry a mortality cost. Radiotherapy, by this logic, treats a compartment—the breast, chest wall and regional nodes—whose failure is more often salvageable and less often lethal in the short term. If one modality must yield priority, it should be the local one. This reasoning is intuitive and, at the population level, broadly sound: the treatment that governs survival should not be made to wait behind the treatment that governs local control [2,6].

The argument is not without tension, however. Large meta-analyses show that locoregional radiotherapy also reduces breast cancer mortality [1], and randomised trials adding post-mastectomy radiotherapy to systemic therapy have demonstrated survival gains beyond local control alone [7,8]. Regional nodal irradiation in MA.20 improved ten-year disease-free survival from 77.0% to 82.0% but did not significantly improve overall survival, which was 82.8% versus 81.8%; we state this distinction explicitly so that the trial is not read as evidence of a survival benefit [9]. The magnitude of the radiotherapy benefit tracks the reduction in local recurrence, implying that uncontrolled local disease can itself seed fatal distant relapse [1]. The clean separation between a “local” modality and a “systemic” one is therefore a simplification. If radiotherapy contributes to systemic control by eradicating a reservoir capable of secondary dissemination, then delaying it is not obviously safer than delaying chemotherapy; it is a different kind of risk, not the absence of one. We concede, however, that this evidence establishes that radiotherapy should be given rather than that it should be given earlier, and it therefore does not by itself answer the quantitative argument set out in Section 3.4.

### 3.2. Delaying Radiotherapy Appears Safe—Within Limits

The convention depends on a second, empirical claim: that postponing radiotherapy to accommodate chemotherapy does not compromise local control, provided the delay is bounded. A Cochrane review of the randomised evidence concluded that different sequences of chemotherapy and radiotherapy did not materially affect recurrence or survival as long as radiotherapy commenced within roughly seven months of surgery [10]. Systematic reviews of waiting times, large registry analyses of radiotherapy timing after breast-conserving surgery, and dedicated reviews of adjuvant timing reach broadly similar conclusions for intervals of up to a few months [11,12,13].

That reassurance has an expiry date. Older series suggest that once radiotherapy is delayed beyond about six months, local recurrence rises and, in some analyses, distant recurrence and survival worsen as well [14], and modern retrospective data likewise find that chemotherapy delayed well beyond twelve weeks after surgery is associated with inferior disease-free survival [15]. A patient who receives an anthracycline followed by a taxane, with the attendant recovery intervals, may not begin radiotherapy until five to seven months after surgery—precisely the region where the evidence for safety becomes thin. The chemotherapy-first rule thus contains an internal limit that modern regimens are capable of breaching, a point to which we return in Section 5. It is important to be clear about the direction in which the second of these findings points: if chemotherapy delayed beyond twelve weeks worsens disease-free survival, that observation tells against upfront radiotherapy rather than for it, since any strategy that postpones systemic treatment inherits precisely this risk. We cite it as a constraint upon the strategies discussed in Section 7, not as support for them.

### 3.3. Overlapping Toxicity and the Decline of Concurrent Chemoradiotherapy

The third argument is pragmatic and concerns toxicity rather than efficacy. Delivering cytotoxic chemotherapy and radiotherapy simultaneously superimposes their acute effects—myelosuppression, mucositis, radiation dermatitis, oesophagitis, pneumonitis and, for anthracyclines and left-sided fields, cardiac risk. Concurrent regimens built on anthracyclines and taxanes have proved more toxic than sequential delivery, and this, as much as any efficacy consideration, is why concurrent chemoradiotherapy never became standard for these agents [2].

The picture is more nuanced for older, gentler regimens. The SECRAB trial, the largest randomised comparison of synchronous versus sequential chemoradiotherapy, enrolled 2297 patients treated predominantly with cyclophosphamide–methotrexate–fluorouracil (CMF) or anthracycline–CMF and reported a genuine reduction in ten-year local recurrence with the synchronous approach, from 7.1% to 4.6%, with the greatest benefit in the anthracycline–CMF subgroup [16]. Synchronous treatment did increase acute skin toxicity, but this was mitigated when radiotherapy was delivered over three weeks rather than longer, leading the investigators to recommend that any concurrent schedule use hypofractionated radiotherapy [16]. The trial was not powered for a survival outcome, and neither disease-free nor overall survival differed significantly between the arms; the benefit demonstrated was confined to local control [16]. An earlier randomised study of sequencing reached broadly concordant conclusions, with the order of treatment influencing the pattern rather than the overall rate of failure [17]. SECRAB is important precisely because it demonstrates that concurrency can improve local control when the cytotoxic partner is tolerable—an existence proof that the objection to concurrent treatment is agent-specific, not absolute.

### 3.4. The Contemporary Rationale: An Asymmetry of Absolute Benefit

The three arguments above are historical and mechanistic. A fourth argument, which does not depend upon the Recht trial and would hold even if that trial had never been conducted, is quantitative, and it is this argument that most convincingly sustains chemotherapy-first sequencing in contemporary practice. It deserves to be stated in its strongest form rather than left implicit.

With modern radiotherapy, locoregional recurrence after breast-conserving surgery in appropriately treated patients runs at approximately five percent at ten years, a figure consistent with the ten-year isolated locoregional disease-free survival of 95.2% reported in the regional nodal irradiation arm of MA.20 [9]. A sequencing change that reduced recurrence from five to three percent would deliver an absolute gain of two percentage points in locoregional control. The relationship established by the EBCTCG, in which approximately one breast cancer death is avoided by fifteen years for every four locoregional recurrences avoided by ten years [1], converts that two-point gain into roughly half a percentage point of absolute mortality benefit. The gain, in other words, passes through a divisor before it reaches the endpoint that matters.

Degradation of systemic therapy carries no comparable divisor. A reduction in chemotherapy dose intensity, or a delay sufficient to compromise it, translates into distant recurrence and death with far less attenuation, because distant relapse is the mortality pathway itself rather than a surrogate for it. The asymmetry is therefore structural rather than incidental: the potential gain from repositioning radiotherapy is small, indirect and discounted, whereas the potential loss from disturbing chemotherapy is larger and direct. This is a considerably stronger argument than the one on which the convention was originally founded, and it is not answered by demonstrating that the historical evidence was weak. Establishing that the Recht trial cannot bear the weight placed upon it leaves the arithmetic untouched.

Candour requires acknowledging what cannot be quantified. There is no randomised estimate of the absolute survival cost of a short, deliberate delay in the initiation of chemotherapy, and none can be derived from the sequencing literature, which was not designed to produce one. What can be offered is non-causal context. In the propensity-matched analysis of Chen et al., patients treated with radiotherapy first, and therefore with later chemotherapy, had an eight-year distant metastasis rate of 14.6% compared with 8.6% in the chemotherapy-first group and a disease-free survival of 83.3% compared with 91.0% [15]. These are observational differences, subject to confounding by indication and to the selection of patients into each sequence, and they cannot be attributed to sequencing as such or read as the expected loss from a planned short delay. These findings illustrate the direction of concern but do not estimate the magnitude or causal effect of sequencing. The ledger therefore places a reasonably characterised potential mortality gain of approximately 0.5 percentage point against an unquantified potential systemic loss.

We accept this asymmetry, and it follows that a departure from conventional sequencing is defensible only under conditions that can be stated explicitly. First, the strategy must not reduce chemotherapy dose intensity or delay its initiation beyond the interval associated with inferior disease-free survival; upfront radiotherapy, which necessarily postpones systemic treatment, must satisfy this requirement before it can be contemplated outside a trial. Second, the expected locoregional gain must be large enough that, after division by the EBCTCG factor, it is not trivially small, which favours populations with higher baseline locoregional risk or with unusually protracted surgery-to-radiotherapy intervals. Third, the absolute benefit of chemotherapy in the population concerned must be genuinely modest so that the quantity placed at risk is correspondingly small. These conditions are restrictive. They define the boundaries of the question rather than dissolving it, and whether any real population satisfies all three is the subject of Section 8.

## 4. Endocrine, Anti-HER2 and Immune Agents: Why They Are Sequenced Differently

The asymmetry that puzzles many clinicians—radiotherapy may accompany endocrine therapy or trastuzumab but not cytotoxic chemotherapy—is explained less by biology than by pharmacology and logistics [2,3]. Three properties distinguish the agents that tolerate concurrency.

The first is the therapeutic index. Endocrine agents such as tamoxifen and aromatase inhibitors have a wide margin between effect and acute harm; they do not appreciably compound the myelosuppressive or mucosal toxicity of irradiation. Cytotoxic chemotherapy, by contrast, is dose-limited by the very toxicities that radiotherapy also produces, so concurrency narrows an already narrow margin. The second is the delivery window. Cytotoxic regimens must be given at full dose and on schedule to preserve efficacy; a competing modality that forces dose reductions or delays is self-defeating. Endocrine therapy, administered continuously over five to ten years, has no comparable schedule to protect—a few weeks of overlap with radiotherapy consumes an inconsequential fraction of its course. The third is duration itself: because endocrine treatment is so prolonged, insisting that it wait for radiotherapy would either delay it by months or interrupt it, neither of which is desirable, so concurrency is simply the path of least resistance.

Anti-HER2 antibody therapy occupies an intermediate position and is generally continued through radiotherapy with attention to cardiac dose, particularly for left-sided disease and internal mammary irradiation. The concurrent use of immune checkpoint inhibitors with radiotherapy, now relevant to triple-negative disease, is under active investigation; the theoretical appeal of radiation-induced immunogenic priming is balanced against uncertain overlapping toxicity, and practice here is not yet settled. The unifying principle is that the prohibition was never really “radiotherapy before chemotherapy is wrong.” It is that radiotherapy should not displace, dilute or collide with the full-dose cytotoxic course on which distant control depends. Agents that neither compete for the same delivery window nor share the same acute toxicities are exempt from the concern.

## 5. When the Evidence Base Outgrew the Convention

### 5.1. Third-Generation Chemotherapy Lengthened Systemic Treatment

The chemotherapy tested in the trial that founded the convention lasted twelve weeks. Contemporary sequential regimens—an anthracycline-based course followed by a taxane, with dose-dense or weekly scheduling and, increasingly, additional agents—commonly occupy four to six months, and the interposed recovery periods extend the interval from surgery to the completion of systemic therapy still further [2]. The “killing time” of adjuvant treatment, the total interval a patient spends under active therapy, has grown substantially, and radiotherapy sits at the end of it.

### 5.2. The Radiotherapy-Delay Paradox

The lengthening of chemotherapy collides directly with the bounded safety of the radiotherapy delay described in Section 3.2. The original evidence that a modest deferral is harmless was generated with short chemotherapy; applied to modern regimens, the same rule can push the start of radiotherapy toward or past the six- to seven-month threshold, beyond which older series suggest local control may suffer [10,14]. The evidence here is not uniform and should not be overstated. A large propensity-matched analysis found that delaying radiotherapy for up to thirty-two weeks in order to deliver long-course chemotherapy did not compromise locoregional control or survival, whereas delaying chemotherapy beyond twelve weeks was detrimental [15]. That study therefore constrains the upfront strategy rather than supporting the case for moving radiotherapy earlier, and we cite it accordingly. The convention thus contains a paradox: adhering strictly to “chemotherapy first” in the era of prolonged systemic therapy may, in some patients, reproduce precisely the radiotherapy delay that older data warned against. This is not an argument against systemic therapy, which is non-negotiable where indicated, but an argument that the sequencing question cannot be answered once and for all with evidence drawn from a different therapeutic era.

## 6. Hypofractionation: Radiotherapy Becomes a Short, Movable Module

**Figure 1:**
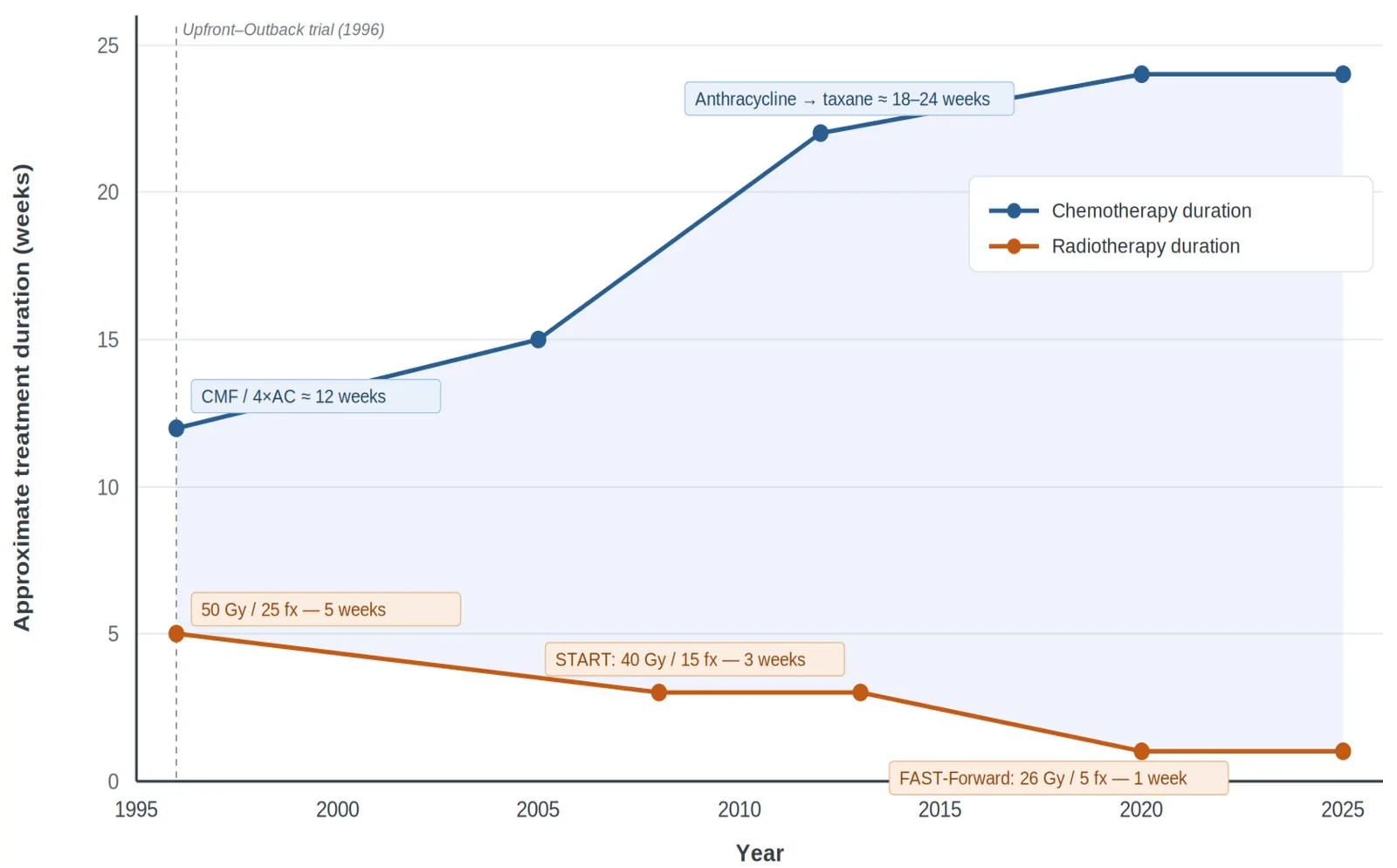
Divergence of adjuvant chemotherapy and radiotherapy durations in early breast cancer, c. 1996–2025. Over three decades, cytotoxic chemotherapy has lengthened from the twelve-week regimen used in the trial that founded the chemotherapy-first convention to the eighteen- to twenty-four-week anthracycline–taxane sequences in current use, while whole-breast radiotherapy has contracted from 50 Gy in 25 fractions over five weeks to 40 Gy in 15 fractions over three weeks and, most recently, 26 Gy in 5 fractions over one week. The widening band between the two curves is the logistical space in which sandwich and upfront radiotherapy have become feasible. Radiotherapy durations shown are for whole-breast irradiation; where a sequential tumour-bed boost is required, approximately one further week should be added. Durations are approximate and shown for schematic purposes.

While chemotherapy lengthened, radiotherapy underwent the opposite transformation (Figure 1). The conventional schedule of 45–50 Gy in 25 fractions over five weeks has been progressively displaced. The UK Standardisation of Breast Radiotherapy (START) trials established that 40 Gy in 15 fractions over three weeks was at least as effective and no more toxic than the conventional regimen, and this moderately hypofractionated schedule became a widely adopted standard [18,19]. The FAST-Forward trial then compressed treatment further, randomising 4096 patients to 40 Gy in 15 fractions or to one-week schedules of 26 or 27 Gy in five fractions; at five years, 26 Gy in five fractions over one week was non-inferior for local control and comparable in late normal-tissue effects to the three-week regimen, and it has since entered guidelines as a standard of care [20]. One qualification should be entered here. The one- and three-week schedules quoted above describe whole-breast irradiation alone. Where a boost to the tumour bed is indicated, as it commonly is for younger patients or those with close margins or other adverse features [21], and where brachytherapy is precluded by anatomical considerations such as tumour bed depth, breast volume or cavity geometry, a sequential external beam boost adds approximately one further week and occasionally more. A simultaneous integrated boost avoids this extension but is neither universally available nor appropriate in every anatomical configuration. The radiotherapy module is therefore short, but not invariably as short as the headline schedules imply, and any intercalation strategy must be planned around the boost requirement of the individual patient rather than around the briefest published regimen.

The implication for sequencing is easily overlooked. A course of radiotherapy that once demanded five to six weeks of a patient’s time and could not plausibly be threaded between chemotherapy cycles can now be delivered, for whole-breast irradiation, in one to three weeks, subject to the boost qualification noted above. Radiotherapy has, in effect, become a short and movable module. The logistical objection that historically made intercalation impractical—that inserting weeks of daily irradiation into a chemotherapy schedule would unacceptably disrupt it—has been substantially reduced. What was once a fixed, immovable block at the end of treatment is now a component that could, in principle, be repositioned. Whether it should be is an empirical question that the field is only beginning to ask.

## 7. Revisiting Sequence: Sandwich and Upfront Radiotherapy

Two strategies exploit the new brevity of radiotherapy: interleaving it between chemotherapy cycles (“sandwich” radiotherapy) and delivering it before chemotherapy altogether (“upfront” radiotherapy). Both remain investigational. The supporting evidence consists of two retrospective series, neither of which demonstrates a benefit in distant control or survival, and both of which carry design limitations that materially qualify their findings, as set out in Section 7.1 and Section 7.2. We therefore present these strategies as hypothesis-generating rather than as established candidates for a change in practice. Table 1 summarises the four principal sequencing strategies, their supporting evidence and their current status.

### 7.1. Sandwich (Intercalated) Radiotherapy

The sandwich concept—radiotherapy delivered between chemotherapy cycles rather than after all of them—has a long history with older regimens, but the most instructive contemporary data come from a retrospective cohort of 200 node-positive patients, all treated with doxorubicin and cyclophosphamide followed by a taxane, in which radiotherapy was given either between the anthracycline and taxane phases or after the entire chemotherapy course [22]. Over a mean follow-up exceeding eight years, the sandwich approach was associated with a markedly lower locoregional recurrence rate, 3.6% versus 13.3%, with no significant difference in distant metastasis, and radiotherapy sequence remained an independent predictor of locoregional control on multivariable analysis [22]. The interval from surgery to radiotherapy fell from a mean of 5.5 to 3.0 months, and in the luminal A subgroup, disease-free survival was superior with the sandwich schedule [22]. One mechanistic reading is that, where chemotherapy confers relatively little benefit, as in luminal A disease, the timing of radiotherapy exerts proportionally greater influence on outcome, so bringing it forward pays a local-control dividend. This interpretation is plausible but should not be adopted before the limitations set out below have been weighed.

These findings should be read with the caution that retrospective, single-institution data demand, and they carry an important agent-specific caveat inherited from earlier work: interrupting an anthracycline course to insert radiotherapy raised historical concerns, and much of the older sandwich experience involved CMF rather than anthracycline-based schedules [22]. The lesson is not that intercalation is universally safe but that, with appropriate regimens and modern short radiotherapy, it is feasible and may improve locoregional outcomes without demonstrably compromising systemic control.

Three limitations of this study require explicit statement, since the argument of this review should not be advanced with more confidence than they permit. First, the control-arm locoregional recurrence rate of 13.3% is substantially higher than would be expected for node-positive disease treated with contemporary radiotherapy, including regional nodal irradiation, where figures of approximately five percent are typical and MA.20 reported ten-year isolated locoregional disease-free survival of 95.2% [9]. A control event rate of this magnitude in a retrospective single-institution series raises the possibility that cohort characteristics, radiotherapy technique or target coverage, or the selection of patients for the sandwich schedule account for a material part of the apparent difference, and the reported effect size should therefore be interpreted with corresponding caution. Second, allocation was not randomised, and the clinical reasoning that led to intercalation in some patients and not others cannot be fully recovered; confounding by indication cannot be excluded. Third, the superior disease-free survival observed in the luminal A subgroup derives from an unplanned subgroup analysis within a retrospective cohort and carries the well-recognised hazards of such analyses. It should also be noted that the study did not formally compare toxicity between the two schedules and that the absence of a difference in distant metastasis does not establish that systemic efficacy was preserved. The finding is suggestive, and it informs the enrichment strategy proposed in Section 10, but it cannot bear the weight of a principal argument, and the claims drawn from it have been moderated accordingly.

### 7.2. Upfront Hypofractionated Radiotherapy

The more radical proposal reverses the sequence entirely. In a retrospective comparative study, 45 patients with node-positive disease received hypofractionated radiotherapy—15 or 5 fractions delivered with intensity-modulated or volumetric-modulated arc techniques, with or without a simultaneous integrated boost—before their third-generation adjuvant chemotherapy and were matched against 45 patients treated in the conventional order [23]. Radiotherapy began a median of about five weeks after surgery, chemotherapy started roughly ten days after its completion, and the entire adjuvant course was compressed, with no significant excess in acute or subacute skin or pulmonary toxicity relative to standard sequencing [23]. The approach was modelled on established hypofractionation protocols, demonstrating that upfront radiotherapy is not merely a theoretical construct but a deliverable schedule; prospective randomised protocols incorporating hypofractionation with a simultaneous integrated boost and regional nodal irradiation further underscore the flexibility of modern delivery [24]. This study should nevertheless be characterised accurately. With forty-five matched pairs and acute and subacute toxicity as its endpoints, it is a feasibility and tolerability report rather than a source of efficacy evidence. It establishes that upfront hypofractionated radiotherapy can be delivered without an evident excess of early toxicity; it does not establish that locoregional control, distant control or survival are preserved, and no such inference should be drawn from it.

The appeal of upfront radiotherapy is twofold. Logistically, it removes radiotherapy from the tail of a lengthening treatment course and thereby addresses the radiotherapy-delay component of the paradox described in Section 5.2: local therapy is delivered promptly, when its benefit is least likely to be eroded by waiting, and systemic therapy follows without having been itself postponed by more than a short interval. Practically, it shortens the overall treatment time and may ease the sequencing burden on patients and departments alike [23].

### 7.3. A Biological Rationale for Early Local Therapy

There is a further, more speculative argument that early local treatment may be actively advantageous rather than merely permissible. Surgery provokes a wound-healing response, and there is experimental interest in the possibility that the perioperative milieu transiently favours the proliferation and dissemination of residual tumour cells; early irradiation of the tumour bed has been hypothesised to blunt this surge [2,23]. Radiotherapy also modulates the local immune environment and can, under some conditions, contribute to systemic tumour control—the basis of ongoing interest in combining irradiation with immunotherapy. These mechanisms remain hypotheses rather than established facts, and they should be presented as such. But they reframe the founding premise of the convention: if early local therapy can suppress rather than merely fail to prevent micrometastatic seeding, then the reflexive assumption that radiotherapy must always yield priority to systemic treatment deserves re-examination rather than automatic deference.

## 8. The Population in Which the Question Remains Clinically Live

An objection that the preceding argument must answer is that the population in which the asymmetry of Section 3.4 is tolerable and the population in which the delay problem actually arises may not be the same population. The objection is well founded, and we state it plainly rather than deflecting it.

The asymmetry becomes tolerable only where the absolute benefit of chemotherapy is small, which is why lower-chemosensitivity disease has featured in the retrospective signals discussed above. Following TAILORx [25] and RxPONDER [26], however, these are precisely the patients increasingly spared chemotherapy altogether. TAILORx established that endocrine therapy alone was non-inferior to chemoendocrine therapy in node-negative disease with an intermediate recurrence score, and RxPONDER showed that postmenopausal women with one to three positive nodes and a recurrence score of twenty-five or lower derive no benefit from adjuvant chemotherapy. Where chemotherapy is not administered, no sequencing question arises. Conversely, the patients who genuinely experience surgery-to-radiotherapy intervals of five to seven months are those receiving full anthracycline and taxane courses for node-positive, triple-negative or high-risk HER2-positive disease, in whom the preservation of dose intensity matters most, and the quantity placed at risk is largest. The delay paradox is thus most acute in the population for which the proposed remedy is least appropriate.

Two further considerations narrow the live population. The exclusion of the neoadjuvant setting, adopted here for coherence, removes a growing share of higher-risk patients who now receive systemic therapy before surgery. And the de-escalation trend discussed in Section 10 operates against the agenda rather than for it, since it withdraws from chemotherapy the very phenotypes with the most favourable sequencing arithmetic. We were also unable to identify any reliable, published estimate of the size of the residual population, and the honest position is that it has not been quantified.

What remains is narrower than the framing of this review might initially suggest, and the difficulty should be stated rather than resolved by assertion. A candidate population would comprise patients who require chemotherapy but in whom its absolute benefit is moderate rather than large, who carry appreciable baseline locoregional risk, and in whom the surgery-to-radiotherapy interval is predictably protracted. Premenopausal women with one to three positive nodes and intermediate recurrence scores are the group most often nominated in this context, but they illustrate the problem rather than solving it: in RxPONDER the five-year invasive disease-free survival difference in this group was 4.9 percentage points, 93.9% with chemoendocrine therapy against 89.0% with endocrine therapy alone [26], which is clinically meaningful and substantially larger than the illustrative half a percentage point computed in Section 3.4. This group does not therefore clearly satisfy the third condition, and we do not present it as a settled candidate population. It remains possible that no population satisfies all three conditions simultaneously, and that possibility should be tested rather than assumed away. Establishing whether an adequate population exists, and quantifying it by registry analysis, is accordingly a precondition of the research agenda set out in Section 10 rather than a detail within it.

## 9. Balancing the Argument

A fair appraisal must give the conservative position its due. The case for retaining chemotherapy-first sequencing does not rest solely on inertia. No randomised trial has yet shown that sandwich or upfront radiotherapy is non-inferior for the endpoints that matter most—distant metastasis and overall survival—in patients treated with contemporary systemic therapy. The supportive data are retrospective, subject to selection bias, and concentrated in favourable subgroups such as luminal A disease. The toxicity of concurrent anthracycline- and taxane-based chemoradiotherapy is real and well documented, and any strategy that risks reproducing it must clear a high safety bar. Full-dose, on-schedule delivery of systemic therapy remains the single most important determinant of survival for many patients, and a sequencing innovation that jeopardises it would be a poor trade even if it improved local control. This concession is not rhetorical. It grants the asymmetry of absolute benefit set out in Section 3.4, and it is the reason we regard upfront radiotherapy in particular as confined to the trial setting: a strategy that postpones systemic therapy places at risk the very determinant just identified as paramount.

The case for reopening the question is equally serious. The convention was founded on a small trial whose central signal did not survive long-term follow-up, using chemotherapy the trialists themselves declined to extrapolate from [4,5]. The lengthening of systemic therapy may, for some patients, push radiotherapy toward intervals associated with poorer local control in older observational series [14], although the evidence on this point is not uniform (Section 5.2). Hypofractionation has substantially reduced the logistical barrier that made intercalation impractical [18,20]. And the two modern series differ in what they support: the sandwich cohort provides a hypothesis-generating locoregional signal, qualified by an unexpectedly high control-arm event rate and by reliance on an unplanned subgroup analysis [22], whereas the upfront series supports short-term feasibility and tolerability alone [23]. Neither demonstrates benefit in distant control or survival. Taken together, these do not prove that the convention is wrong, and they do not overturn the arithmetic of Section 3.4, which continues to favour it in most populations. What they establish is narrower: that the convention is no longer self-evidently right, and that within a defined and possibly contracting population its central premise is a hypothesis amenable to, and deserving of, prospective test.

## 10. Future Directions

The obvious deficiency in this field is the near-total absence of randomised evidence generated in the modern therapeutic era. Adequately powered trials comparing conventional chemotherapy-first sequencing against sandwich and upfront hypofractionated schedules, with distant metastasis and survival as primary endpoints and rigorous toxicity and quality-of-life capture, are needed before practice can responsibly change. Such trials should be enriched for the subgroups in which the biological rationale is strongest, namely lower-chemosensitivity phenotypes in which the timing of local therapy may carry disproportionate weight [22]; the evidence for this enrichment strategy rests on an unplanned subgroup analysis and is correspondingly provisional. A prior requirement, discussed in Section 8, is a registry-based estimate of the size of the population in which the question remains clinically live. Designing a randomised trial before establishing that an adequate population exists would be premature. Ongoing work on hypofractionated schedules incorporating simultaneous integrated boost and regional nodal irradiation, and on preoperative radiotherapy, will further clarify how flexibly modern radiotherapy can be positioned within a multimodal course [20,24].

Two broader currents bear on this agenda, and they do not run in the same direction. The first is capacity: ultra-hypofractionation substantially reduces the resource cost of radiotherapy, and sequencing strategies that shorten overall treatment time have value to health systems as well as to patients. The second is de-escalation, which we previously offered as evidence that the agenda is timely and now state with the opposite emphasis. Genomically guided de-escalation removes from chemotherapy precisely those phenotypes in which the sequencing arithmetic is most favourable, so the trend in systemic therapy narrows rather than widens the constituency for this question. A question once regarded as closed is worth reopening, but for a defined population whose size has been established rather than assumed.

## 11. Conclusions

The instruction that radiotherapy should follow chemotherapy in adjuvant breast cancer is a reasonable default that has hardened into doctrine. Its foundations—a borderline and non-replicated distant-metastasis signal, an intuitive but incomplete separation of “local” from “systemic” control, and a genuine concern about overlapping cytotoxic toxicity—were laid with short chemotherapy and long radiotherapy. Both of those conditions have since reversed. Chemotherapy has lengthened to the point where strict adherence may push radiotherapy toward intervals associated with poorer local control in older observational series, while radiotherapy has shrunk to a one- to three-week whole-breast schedule, potentially extended when a sequential tumour-bed boost is required, which can be intercalated or delivered upfront with far less logistical disruption than such approaches once entailed. The retrospective experience is more limited than it may appear, and the two series support different things: a sandwich cohort provides a hypothesis-generating locoregional signal, qualified by an unexpectedly high control-arm event rate and an unplanned subgroup analysis, while an upfront series supports short-term feasibility and tolerability alone. Neither demonstrates benefit in distant control or survival. Two constraints must be carried alongside this conclusion. The asymmetry of absolute benefit continues to favour chemotherapy-first sequencing wherever the benefit of chemotherapy is substantial, and the population in which the question remains clinically live is narrow and may be contracting as genomic de-escalation advances. The prudent conclusion is neither to abandon the convention nor to defend it reflexively, but to treat adjuvant sequencing in breast cancer for what it has quietly become within a defined population: an open and testable question, whose answer should be sought in a randomised trial designed for the patients in whom it still arises.

## Figures and Tables

**Table 1:** Comparison of adjuvant chemotherapy–radiotherapy sequencing strategies in early breast cancer. Abbreviations: RT, radiotherapy; RCT, randomised controlled trial; JCRT, Joint Center for Radiation Therapy; CMF, cyclophosphamide–methotrexate–fluorouracil.

Approach	Sequence and Timing	Key Evidence	Efficacy and Toxicity Signal	Status
Sequential (chemotherapy → radiotherapy)	RT after completion of all chemotherapy; RT ideally within ~7 months of surgery	JCRT “Upfront–Outback” RCT, n = 244 [4,5]; Cochrane review [10]	Reference standard; local control preserved if RT not unduly delayed; lowest overlapping toxicity	Guideline standard of care
Concurrent (synchronous)	RT delivered during chemotherapy	SECRAB RCT, n = 2297 (CMF or anthracycline–CMF) [16]	10-year local recurrence 7.1% → 4.6%; increased acute skin toxicity, mitigated by ≤3-week RT; anthracycline–taxane concurrency more toxic	Acceptable for selected (non-taxane) regimens
Sandwich (intercalated)	RT inserted between anthracycline and taxane phases	Retrospective node-positive cohort, n = 200 [22]	Lower locoregional recurrence in a retrospective cohort (3.6% vs. 13.3%); no significant difference in distant metastasis; luminal A disease-free survival signal from an unplanned subgroup analysis; toxicity not formally compared	Investigational
Upfront (radiotherapy first)	Hypofractionated RT before chemotherapy	Retrospective matched cohort, n = 45 [23]	Short-term feasibility and tolerability only; compressed overall treatment time; no excess acute or subacute toxicity; no efficacy or survival data	Investigational

## Data Availability

No new data were created or analysed in this study. Data sharing is not applicable to this article.
